# COVID-19 and Its Potential Impact on Children Born to Mothers Infected During Pregnancy: A Comprehensive Review

**DOI:** 10.3390/diagnostics14212443

**Published:** 2024-10-31

**Authors:** Cristiana Stolojanu, Gabriela Doros, Melania Lavinia Bratu, Iulia Ciobanu, Krisztina Munteanu, Emil Radu Iacob, Laura Andreea Ghenciu, Emil Robert Stoicescu, Mirabela Dima

**Affiliations:** 1 Doctoral School, ‘Victor Babes’ University of Medicine and Pharmacy, 300041 Timisoara, Romania; cristiana.stolojanu@umft.ro; 2 ‘Louis Turcanu’ Emergency Hospital for Children, 300011 Timisoara, Romania; doros.gabriela@umft.ro; 3 Department of Pediatrics, ‘Victor Babes’ University of Medicine and Pharmacy, 300041 Timisoara, Romania; 4 Center for Neuropsychology and Behavioral Medicine, Discipline of Psychology, Faculty of General Medicine, ‘Victor Babes’ University of Medicine and Pharmacy Timisoara, 300041 Timisoara, Romania; 5 Center for Cognitive Research in Neuropsychiatric Pathology, Department of Neurosciences, ‘Victor Babes’ University of Medicine and Pharmacy Timisoara, 300041 Timisoara, Romania; 6 Department of Anatomy and Embriology, ‘Victor Babes’ Univeristy of Medicine and Pharmacy, 300041 Timisoara, Romania; ciobanu.iulia@umft.ro (I.C.); krisztina.munteanu@umft.ro (K.M.); 7 Department of Pediatric Surgery, ‘Victor Babes’ University of Medicine and Pharmacy, 300041 Timisoara, Romania; radueiacob@umft.ro; 8 Department of Functional Sciences, ‘Victor Babes’ University of Medicine and Pharmacy Timisoara, 300041 Timisoara, Romania; bolintineanu.laura@umft.ro; 9 Radiology and Medical Imaging University Clinic, ‘Victor Babes’ University of Medicine and Pharmacy Timisoara, 300041 Timisoara, Romania; stoicescu.emil@umft.ro; 10 Research Center for Pharmaco-Toxicological Evaluations, ‘Victor Babes’ University of Medicine and Pharmacy Timisoara, 300041 Timisoara, Romania; 11 Research Center for Medical Communication, ‘Victor Babes’ University of Medicine and Pharmacy Timisoara, 300041 Timisoara, Romania; 12 Field of Applied Engineering Sciences, Specialization Statistical Methods and Techniques in Health and Clinical Research, Faculty of Mechanics, ‘Politehnica’ University Timisoara, 300222 Timisoara, Romania; 13 Department of Neonatology, ‘Victor Babes’ University of Medicine and Pharmacy, 300041 Timisoara, Romania; dima.mirabela@umft.ro

**Keywords:** maternal COVID-19 effects, fetal development, placental dysfunction, child development

## Abstract

Pregnancy is a vulnerable period of time during which pregnant people are prone to infections like COVID-19, which can increase risks for both the mother and fetus. These infections may lead to complications such as preterm birth, developmental delays, and congenital abnormalities. While COVID-19 poses additional risks like placental dysfunction and neonatal infections, studies on long-term effects remain limited. Ongoing research and monitoring are essential to understand and mitigate potential cognitive and developmental challenges in children born to mothers infected with COVID-19. This review aims to guide clinicians in managing these risks throughout childhood. Maternal COVID-19 infection during pregnancy can have significant implications for fetal development, even if the newborn is not infected at birth. The release of inflammatory cytokines may cross the placental barrier, potentially disrupting fetal brain development and increasing the risk of long-term cognitive and behavioral issues, such as ADHD or autism. Placental dysfunction, caused by inflammation or thrombosis, can lead to intrauterine growth restriction (IUGR), preterm birth, or hypoxia, affecting both neurological and respiratory health in newborns. Furthermore, a compromised fetal immune system can increase susceptibility to autoimmune conditions and infections. The early diagnosis and management of infections during pregnancy are crucial in mitigating risks to both the mother and fetus. Swift intervention can prevent complications like preterm birth and long-term developmental challenges, ensuring better health outcomes for both the mother and child. Long-term monitoring of children born to mothers infected with COVID-19 is necessary to understand the full extent of the virus’s impact. This review evaluates the long-term systemic effects of maternal COVID-19 infection during pregnancy on fetuses, newborns, and children, focusing beyond vertical transmission. It highlights the broader impacts on fetal development, offering insights to help clinicians manage potential issues that may arise later in life.

## 1. Introduction

Pregnancy is divided into three trimesters, with the placenta playing a vital role early on. In the first trimester (weeks 1–12), the placenta forms from trophoblast cells, establishing the essential mother–fetus connection [1,2]. It facilitates the exchange of oxygen, nutrients, and waste. In the second trimester (weeks 13–26), it grows and matures, supporting rapid fetal growth. By the third trimester (weeks 27–40), the placenta is fully functional, aiding lung and brain development as the fetus prepares for birth. Each trimester plays a key role in fetal development, with the placenta ensuring sustenance throughout [2].

Pregnancy weakens the immune system, increasing infection risks that may pass to the fetus, potentially causing preterm birth, congenital issues, or developmental problems [3,4]. This dual vulnerability, both the increased susceptibility of the mother and the risk to the fetus, can create a complex clinical scenario for healthcare providers, also causing stress and anxiety for the future mother and her family. It is also well known that pre-existing maternal conditions, such as diabetes, or other metabolic diseases, can significantly worsen the effects of infections like COVID-19 during pregnancy [5,6]. These conditions not only increase the mother’s risk of severe illness but can also amplify complications for the fetus [5].

Managing infections during pregnancy is challenging, as common medications may not be safe for the fetus and can cause complications depending on the trimester [7,8]. Healthcare providers must keep a balance between effectively treating the mother and minimizing any potential harm to the unborn child. This adds significant stress to both medical professionals and the pregnant woman, as decisions around treatment often involve difficult trade-offs [9]. Complications arising from infections, such as maternal sepsis or fetal distress, can exacerbate this stress further [10]. Additionally, the fear of potential long-term consequences for the child adds to the emotional burden on the pregnant woman, heightening her anxiety [7,11,12].

Furthermore, the risk of severe outcomes from infections, such as premature delivery, neonatal complications, or maternal morbidity, underscores the importance of careful monitoring and swift intervention [13]. Managing infections during pregnancy increases pressure on healthcare providers, requiring a multidisciplinary approach from obstetricians, infectious disease specialists, and pediatricians to ensure the safety of both the mother and child [14].

There are numerous studies focused on the potential effects of maternal infections with various viruses during pregnancy, as these infections can have serious consequences for the newborn and child, and can also cause the death of the fetus [15]. Viruses like rubella, cytomegalovirus (CMV), and Zika have been particularly well studied due to their capacity to cross the placental barrier and directly impact fetal development [16,17]. Maternal rubella infection in the first trimester can cause congenital rubella syndrome, leading to severe birth defects like heart abnormalities, hearing loss, and developmental delays. Cytomegalovirus, a common cause of congenital infections, may result in hearing loss, vision impairment, and intellectual disabilities [17]. Zika virus, on the other hand, has been linked to microcephaly. This can lead to severe neurological disabilities and developmental delays [16]. These examples highlight the critical importance of studying the effects of viral infections during pregnancy, as they can cause long-term physical and cognitive impairments in children [18]. Understanding the timing and mechanisms of infections helps researchers and healthcare providers better manage and prevent risks during pregnancy.

COVID-19 is a very contagious disease caused by the SARS-CoV-2 virus, which was first detected in late 2019 in Wuhan, China. As it is well known, it rapidly spread worldwide, leading to a global pandemic [19,20,21]. The virus spreads through respiratory avenues, giving mild to severe symptoms. The severe symptomatology affects particularly older people and people with pre-existing conditions, such as immunocompromised patients [19,22,23]. Many of these people suffered complications such as respiratory failure, from which some of them developed long-COVID-19, a syndrome characterized mostly by fatigue but with remaining pulmonary changes, that became chronic [24,25,26]. Significant psychological repercussions associated with COVID-19, including stress, anxiety, and depression, affected not only those who contracted the virus—due to symptoms, disease progression, and hospitalizations—but also the general population facing the broader disruptions and uncertainties caused by the pandemic [27].

Due to the unique physiological and biological complexities of pregnancy, pregnant women and newborns infected with COVID-19 have received special care and attention from medical teams [28]. It is known that the immune system of pregnant women undergoes significant changes and adjustments to protect the fetus, but these changes can increase their susceptibility to severe infections [29]. COVID-19 risks maternal health and fetal development, potentially causing preterm birth and placental dysfunction, affecting fetal growth [28]. Moreover, newborns infected at birth have immature immune systems, making them particularly vulnerable to severe complications and subsequent infections [30,31].

As of 2024, the COVID-19 pandemic continues to have lasting health impacts, including the widespread issue of the above-mentioned long-COVID syndrome.

Extensive research has focused on COVID-19’s long-term effects in adults, but studies on neonates or infants born to mothers infected with the virus are rare. Understanding these effects is essential for neonatal and pediatric care, helping healthcare providers anticipate risks and improve maternal–fetal health knowledge. Long-term monitoring of these children is crucial to identify potential developmental, cognitive, or behavioral issues that may emerge later in life, shaping better healthcare strategies for their future. This review comprehensively examines the broad systemic effects of maternal COVID-19 infection during pregnancy on fetal, neonatal, and childhood development. Unlike previous reviews focused on infections near birth, our study delves into long-term developmental impacts stemming from maternal infection at any pregnancy stage, independent of newborn infection. By addressing systemic changes such as immune dysregulation, vascular issues, and hormonal alterations, we highlight indirect pathways through which maternal COVID-19 can influence fetal development and potentially lead to delayed effects into adolescence. Our multi-sectional approach captures the complex interactions between maternal health and fetal development, offering clinicians and researchers a more thorough framework to anticipate and manage the potential long-term risks associated with prenatal COVID-19 exposure.

## 2. Material and Methods

**Figure 1:**
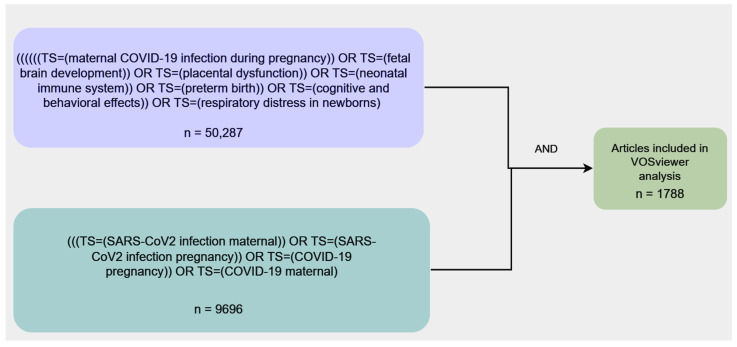
Algorithm for the literature search.

In order to conduct this literature review, two experts with competence in the neonatal and pediatric field evaluated the most relevant literature from the past four years (March 2020 to May 2024) using the PubMed, Scopus, Web of Science, Cochrane, and Google Scholar databases. The selection of the articles was made according to SANRA (Scale for the Assessment of Narrative Review Articles) guidelines [32]. We employed the following MeSH terms for the PubMed search: ((“COVID-19” [MeSH Terms]) OR (“fetal brain development” [MeSH Terms])) OR ((“pregnancy complications, infectious” [MeSH Terms]) OR (“placental dysfunction” [MeSH Terms]). For the Scopus search, we used the topic option to conduct the analysis. The queries targeted the title, abstract, keywords plus, and author keyword fields, applying keywords such as “maternal COVID-19 infection during pregnancy”, “fetal brain development”, “placental dysfunction”, “neonatal immune system”, “preterm birth”, “cognitive and behavioral effects”, and “respiratory distress in newborns.” The same steps and keywords were also used for Web of Science, Cochrane, and Google Scholar. The algorithm for the literature search is represented in the figure below (Figure 1).

From the search results, only the articles that were published in English were selected. After this selection, the most relevant articles for our study were chosen by carefully reading the titles and abstracts. Duplicate articles were removed, as well as articles that were not available for reading and articles that talked only about perinatal maternal COVID-19.

Based on the clustering in the VOSviewer map (version 1.6.20, Leiden, The Netherlands) [33] and the connections between key terms, the research directions can be broken down as follows:

Impact on Neurological Development:

- Cluster (blue) focuses on terms like “brain”, “neurodevelopment”, and “immune activation”. This cluster suggests a concentration of studies investigating how maternal COVID-19 infection affects brain development in fetuses and infants, potentially leading to long-term neurological or developmental conditions.

Impact on the Immune System:

- Cluster (purple) relates to terms such as “immune system”, “T cells”, “cytokines”, and “immune response”. Studies in this area explore the effect of COVID-19 on the neonatal immune system, including how maternal infection may influence neonatal immunity and susceptibility to infections.

Impact on Placenta and Placental Dysfunction:

- Cluster (orange) includes terms like “placenta”, “placental pathology”, and “placental dysfunction”. This research direction looks into how the SARS-CoV-2 virus affects placental function, which is crucial for fetal development and pregnancy outcomes.

Impact on Long-term Cognitive and Behavioral Effects:

- Cluster (could overlap with the neurological development cluster and preterm birth and birth complications) deals with terms like “cognitive effects”, “behavioral effects”, and “long-term outcomes”. This area investigates potential developmental delays or behavioral changes in children whose mothers were infected with COVID-19 during pregnancy.

Impact on Preterm Birth and Birth Complications:

- Cluster (red) revolves around terms such as “preterm birth”, “preterm delivery”, “birth outcomes”, and “birth complications”. This research direction focuses on how maternal COVID-19 infection contributes to preterm labor, low birth weight, and other delivery-related complications.

Impact on Newborn Adaptation—Respiratory Distress Syndrome (RDS):

- Cluster (green) may include terms like “neonatal outcome”, “respiratory distress”, and “pneumonia”. This cluster addresses the respiratory challenges that newborns face, particularly the increased risk of RDS due to maternal infection, and how COVID-19 impacts the adaptation of newborns immediately after birth.

Map Interpretation:

- The central terms like “COVID-19”, “pregnancy”, “SARS-CoV-2 infection”, and “preterm birth” serve as connectors between the different clusters, highlighting their relevance across various study areas.
- The peripheral terms focus more on specific outcomes (e.g., “placental dysfunction” and “immune response”) and represent more targeted investigations within these broad themes.

**Figure 2:**
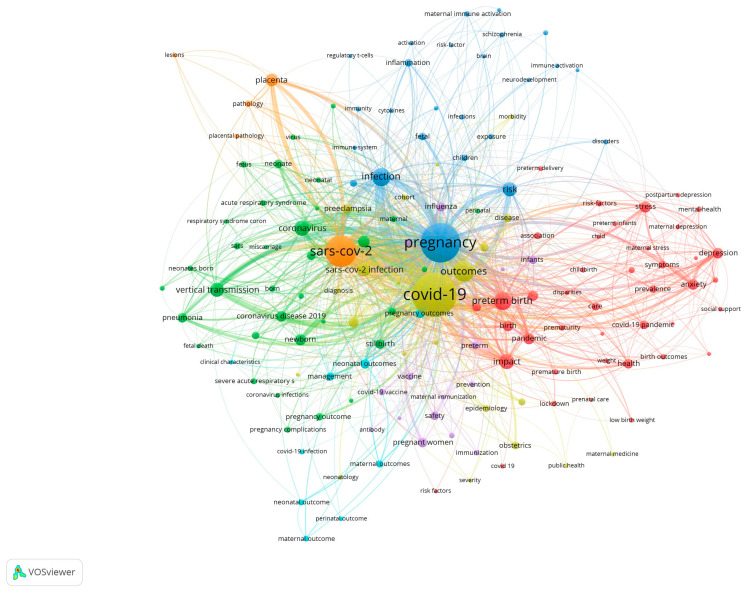
VOSviewer map and the study directions.

The map can be found in the figure below (Figure 2).

The structure of this review intentionally incorporates multiple sections to capture the complex, interrelated maternal health changes and their potential developmental ramifications, thereby offering a foundational basis for clinicians and researchers to better understand and mitigate the long-term risks associated with in utero exposure to maternal COVID-19 infection.

## 3. The Impact of Maternal COVID-19 Infection on the Neurological Development of the Newborn and Infant

COVID-19 infection of the mother during pregnancy, even if the newborn is not positive at birth, can have some serious implications for the neurological development of the newborn [34,35,36]. One of the concerns that was studied by others refers to the maternal immune response to the virus, which can involve the release of inflammation cytokines [37]. It is well known that some of these cytokines are able cross the placental barrier and reach the fetus and its brain, which can lead to altered neurodevelopment, depending on the trimester in which the mother was infected. Studies have shown that inflammation can interfere with white matter development in the fetal brain [34,38].

Even if there are no studies on older children who were born after this kind of maternal infection, it is well known from other kinds of infections that children can later face cognitive and behavioral changes that may manifest later in childhood or adolescence [39]. Some examples of these changes can include delays in language acquisition, motor skills, and increased risk of neurodevelopmental disorders such as autism spectrum disorder (ASD) or attention-deficit/hyperactivity disorder (ADHD) [36].

Nevertheless, there was no direct link found between maternal COVID-19 infection and fetal or neonatal neurological outcomes, which still remains unproven, though several mechanisms suggest this possibility. COVID-19 can induce maternal inflammation and placental dysfunction, potentially impacting fetal brain development. Evidence from other infections, like Zika and rubella, shows similar effects. Preliminary studies suggest that inflammation and placental changes may influence fetal health, but further research is needed [40].

## 4. The Impact on the Immune System of the Newborn and Infant

The immune system of a newborn is developed with the help of the maternal environment during pregnancy. Studies show that if a future mother contracts COVID-19 during pregnancy, this environment can be altered, which can cause effects on the fetal immune system [41,42]. This can happen the same way that was described above, by the passing of inflammatory markers and cytokines from the mother to the fetus through the placental barrier [37,41,43]. 

This way, the fetal and later on newborn’s immune system might become hyperactive, which can trigger autoimmune diseases in childhood or adolescence [44]. On the other hand, if the immune system is poorly developed due to the maternal infection, the newborn and infant may become more vulnerable to infections, not only in the neonatal period but also in the long term [44,45].

## 5. The Impact on the Placenta: Placental Dysfunction

Studies show that one of the issues associated with COVID-19 is placental inflammation [34]. This pathology is characterized by the infiltration of immune cells and cytokines in the placental tissue but also gives a higher risk of placental thrombosis [42,46,47]. This can reduce the ability of the placenta to properly deliver oxygen and nutrients to the fetus [48]. Due to this, the fetus can suffer, which can be manifested through intrauterine growth restriction (IUGR) or even abortion or death [49]. The low oxygen that gets to the fetus can result in hypoxia, which can also alter brain development, making the fetus at risk for future neurological complications.

Furthermore, placental dysfunction can lead to maternal complications, which can also impact the fetus, such as preeclampsia [34,50,51]. This can induce preterm delivery, which itself carries risks of respiratory, neurological, and developmental issues for the newborn or even death of the infant in utero [50]. 

Table 1 shows the summary of placental dysfunction types.

## 6. The Impact on Long-Term Cognitive and Behavioral Effects in Newborns and Infants

Studies show that newborns and infants born from mothers who contracted COVID-19 during pregnancy, even if they were not infected at birth, may face long-term cognitive and behavioral effects [52,53].

As stated before, the exposure of the fetus to inflammatory cytokines, especially during critical periods of brain formation, may lead to alterations in brain structure and function. As a result, these newborns and infants might be at an increased risk of experiencing cognitive delays later in childhood, such as difficulties with memory, attention, and problem-solving skills, which could become apparent as they grow older and enter school [53,54].

Regarding behavior, research on maternal infections during pregnancy, including studies on other viral infections, has shown associations with an increased risk of behavioral disorders such ASD and ADHD [53]. Children born from mothers exposed to COVID-19 during pregnancy might show signs of behavioral difficulties, such as hyperactivity, impulsiveness, or social challenges, which could affect their interaction ability or academic performance [38].

The stress of a mother during pregnancy can have an important effect on the behavior of the future child. Studies show that stress is linked to an increased risk of anxiety and mood disorders in children [55,56].

## 7. The Impact on Birth: Preterm Birth and Complications During Birth

As studies show, maternal infection with COVID-19, even if not present at birth, has been associated with a higher risk of preterm delivery, which can have profound implications for a newborn’s neurological development [35,42]. It is well known that the earlier a baby is born, the higher the risk of complications.

As it is known, during the last trimester of a pregnancy, the fetal brain has rapid growth and development, especially the cerebral cortex, which is responsible for important functions (memory, attention, critical thinking, and problem solving) [57]. When a newborn is born prematurely, this developmental process is interrupted and can result in important cognitive delays, learning disabilities, and difficulties with executive functioning later on in childhood [57].

Preterm infants are also more vulnerable to other neurological complications, such as intraventricular hemorrhage (IVH) [58,59]. Additionally, preterm infants are at an increased risk of developing periventricular leukomalacia (PVL) [57].

## 8. The Impact on the Adaptation of the Newborn: Respiratory Distress Syndrome (RDS)

RDS is a significant concern for newborns, especially preterm infants, and its pathology is characterized by insufficient surfactant production in the lungs, leading to difficulty breathing [60]. Maternal COVID-19 infection can increase the risk of preterm birth, which gives higher risks of RDS development [61,62]. The inflammation associated with the mother’s infection while the fetus is in utero might also compromise lung development in the fetus, which can raise the risk of respiratory complications at birth and later on in childhood [62].

Another respiratory alteration, less severe than RDS, that has been identified in studies is transient tachypnea of the newborn (TTN) [63]. It is often seen in newborns of mothers who experienced infections during pregnancy, including but not limited to COVID-19, as well as other conditions like maternal diabetes or cesarean delivery [63]. Although TTN is generally less severe than RDS, it still requires monitoring and management, as it can be linked to infections that impact fetal lung development [63]. This condition underscores the importance of carefully observing respiratory health in newborns exposed to maternal infections.

Table 2 explains the impacts of maternal COVID-19 infection in newborns and children.

**Figure 3:**
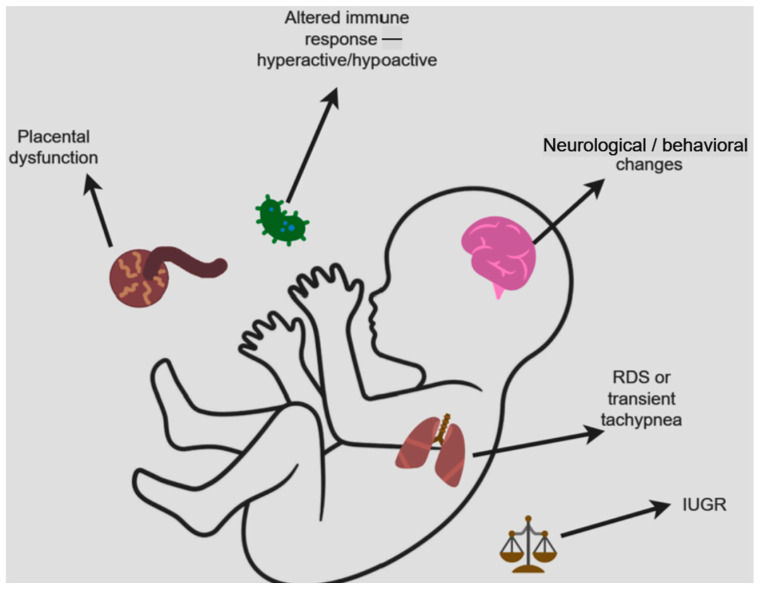
The main possible repercussions of maternal COVID-19 in the newborn.

The figure below (Figure 3) summarizes the impact of maternal COVID-19 on the newborn.

## 9. Discussion

The global COVID-19 pandemic caused the development of significant challenges in understanding its full impact on maternal and child health. While most studies on this topic have focused on the immediate risks and pathologies of pregnant women and their newborns, there are studies that suggest that the implications can extend far beyond birth, and unfortunately can affect the newborn and infant in the long term. Additionally, many of the studies and literature reviews on the effects of maternal COVID-19 infection during pregnancy were conducted at the onset of the pandemic. As a result, these early studies may not provide definitive conclusions, especially regarding long-term effects on children. Given the relatively short observation periods, these studies might not capture potential delayed outcomes, such as cognitive, behavioral, or neurological issues that could manifest after a latent period in which the child’s development appears normal.

As seen in many studies, there is concern regarding the immune system development in newborns and infants from mothers who contracted COVID-19 during pregnancy. The maternal immune response to the virus happens mostly by the production of inflammatory cytokines, which, as studies show, can cross the placental barrier [37,41,44]. This can lead to the disruption of the normal development of the fetal immune system, predisposing the infant to a variety of immune-related outcomes [42,43]. First of all, the infant might develop a hyperreactive immune system, with an increased risk of autoimmune conditions that could manifest either in childhood or even in adulthood in some cases, for example type 1 diabetes, rheumatoid arthritis, or lupus [41,44]. On the contrary, the infant may develop an underactive immune response, making the newborn or child more susceptible to infections [45]. This immune pathology could also impact the child’s response to vaccines [64].

Another issue related to the maternal infection is placental dysfunction, which can lead to many kind of pathologies for the newborn [47]. COVID-19 infection can cause inflammation and thrombosis within the placenta, leading to a reduced ability of the placenta to supply oxygen and nutrients to the fetus [34,46]. This can result in IUGR, low birth weight, hypoxia, and affect fetal brain development [65]. IUGR and hypoxia, in particular, are known to be associated with long-term neurological complications such as cognitive delays and motor dysfunction [54,57,59]. Also, placental dysfunction can lead to preterm labor. Although the fetus has a brain-sparing mechanism that prioritizes blood flow to the fetal brain during low-oxygen conditions, prolonged or severe placental dysfunction can overwhelm this protection, potentially impairing brain development and affecting long-term neurodevelopment [66]. Due to this fact, preterm newborns are at a higher risk of developing IVH and PVL, which can lead to cerebral palsy, cognitive delays, and learning difficulties [34,35,36,52].

Any maternal infection contracted by the mother during pregnancy can potentially lead to complications for her and also for the newborn, one of which is RDS [62]. This condition occurs when the fetus’ lungs are underdeveloped or affected, often due to a lack of surfactant, a substance that, starting seconds after birth, helps to keep the lungs open for proper breathing [67]. Maternal infections, particularly when occurring in the later stages of pregnancy, can trigger an inflammatory response or a placental dysfunction that can interfere with the lung development of the fetus [68]. Subsequently, if a pregnant woman contracts an infection, such as COVID-19, the risk of preterm birth increases, and preterm birth is a main risk factor for RDS [61,69]. Early identification and intervention are crucial to managing RDS and reducing its long-term impact on the child’s health [70].

During the Omicron wave, newborns of unvaccinated mothers experienced significantly higher risks of severe health outcomes, including increased rates of neonatal death and preterm birth, compared to infants born to vaccinated mothers. Research shows that when mothers receive a COVID-19 vaccine or booster within 14 weeks before delivery, they reduce their risk of preterm labor and adverse neonatal events while also transferring COVID-19 antibodies to their babies. This timing supports the baby’s immune defenses in their first weeks of life, providing a layer of protection when their immune system is most vulnerable [71]. Promoting COVID-19 vaccination among women of reproductive age is therefore critical—not only to protect maternal health but also to improve neonatal outcomes in any future COVID-19 surges. As we advance vaccine awareness and access, the focus on timely vaccination before and during pregnancy can help reduce the risk of serious complications for both mothers and newborns, offering substantial benefits in communities affected by ongoing COVID-19 transmission [72].

In addition to the physiological impacts of maternal COVID-19 on the newborn and child, the psychological stress experienced by the mother during pregnancy is known to also play a role in the child’s long-term development [73]. The impact of any kind of infection during pregnancy can have a significant role in high anxiety and stress levels. Research has shown that maternal stress during pregnancy can have a lasting impact on the child’s emotional and behavioral health, increasing the risk of anxiety, mood disorders, and difficulties with emotional regulation. This is especially concerning in the context of COVID-19, where maternal stress may have been exacerbated by factors such as isolation, uncertainty, and fear of infection. The stress caused by infections such as COVID-19, as well as other viral illnesses, can significantly impact the mental and emotional well-being of pregnant women and their support networks [74]. The uncertainty around potential complications for both the mother and child increases anxiety, making it essential for these women to have access to psychological counseling or support groups [75]. These resources can provide emotional relief and a platform to share concerns, reducing the feeling of isolation. Additionally, it is crucial for healthcare providers to explain the potential risks associated with infections to the mother, especially as she approaches childbirth [76]. This includes educating her on what to monitor in the newborn, such as developmental milestones, breathing difficulties, or signs of infection, so that any complications can be identified early and addressed promptly. By offering both emotional and practical support, healthcare providers can help minimize the long-term effects of these complications and promote the well-being of both the mother and child.

However, there is still little evidence on the long-term consequences of COVID-19, as the disease is still relatively new. As these children grow, especially during critical periods like adolescence and young adulthood, when hormonal changes are significant, it will be essential to monitor them for any delayed or subtle impacts that may happen.

## 10. Limitations of the Study

As limitations of the study, we can first mention the lack of sufficient articles that specifically address this topic. Additionally, there is a shortage of long-term studies on children born to mothers infected with COVID-19 during pregnancy. Another limitation is the absence of studies that illustrate the outcomes for newborns and infants based on the trimester in which the mother was infected during pregnancy. Since COVID-19 is a relatively new disease, there may not be enough long-term follow-up data available to assess the full spectrum of developmental, cognitive, or behavioral outcomes in children.

Another limitation of this review is the lack of definitive studies confirming the neurological outcomes of maternal COVID-19 infection on fetal or neonatal development.

Lastly, we believe that another important weakness of our study is the fact that factors such as pre-existing maternal conditions (e.g., hypertension and diabetes) or social determinants of health (e.g., access to healthcare and socioeconomic status) may confound the results and make it difficult to isolate the impact of COVID-19 on pregnancy outcomes.

## 11. Future Directions

For a better understanding of this pathology and its impact on children born to mothers infected with COVID-19 during pregnancy, long-term studies and careful observation of these children are absolutely necessary, not only in the immediate postnatal period or in the first year but also throughout adolescence and young adulthood. Only through this approach can the true impact of maternal infection with COVID-19 on children be understood.

Additionally, it would be beneficial to conduct studies that clearly demonstrate the various complications of the virus depending on the trimester in which the mother contracted the infection.

In the future, it is important to implement educational programs for future mothers in order to raise awareness about the potential long-term effects of COVID-19 on their children, especially if the infection was contacted during pregnancy. These programs should emphasize the need for mothers to be vigilant throughout childhood and adolescence, monitoring their children for any signs of developmental, immunological, cognitive, or behavioral issues that could arise. Encouraging mothers to stay informed and proactive would help ensure the early detection of symptoms, leading to timely interventions that could mitigate complications. By providing clear guidance on what symptoms to watch for, such programs could empower mothers to take an active role in safeguarding their children’s health.

## 12. Conclusions

In conclusion, while the immediate effects of maternal COVID-19 infection are clear and well known, the long-term consequences for children exposed in utero to this virus are still not completely known. The inflammatory response to the virus, both from the mother and the placenta, may cause significant alterations to fetal brain development, leading to long-term issues such as learning disabilities, attention disorders, and a heightened risk for conditions like ADHD and autism. Moreover, the immune system of these children could be compromised, either becoming hyperactive and prone to autoimmune diseases or underdeveloped, leaving them vulnerable to infections and possibly affecting their response to vaccines. Additionally, placental dysfunction, which can result in intrauterine growth restriction and hypoxia, raises the risk of developmental delays and neurological impairments, especially in cases of preterm birth, as well as the development of RDS.

Long-term studies are essential to fully understand the potential impacts of maternal COVID-19 on children, as many of these effects could emerge later in childhood or adolescence. This highlights the importance of continuous research and monitoring over time.

The early diagnosis and management of COVID-19 or any infection during pregnancy are essential for minimizing risks to both the mother and fetus. Prompt treatment can significantly reduce complications such as preterm birth, fetal growth restriction, or developmental issues. Early intervention helps control infection severity in the mother and protects the fetus by preventing placental dysfunction and reducing inflammation. By addressing the infection quickly, the chances of long-term health complications for both the mother and newborn are significantly lowered, ensuring better outcomes for both.

## Figures and Tables

**Table 1:** Short explanation of the placental dysfunction types and their possible outcomes.

Placental Dysfunction	Mechanism: Maternal Infection	Possible Outcomes for Fetus or Newborn
Placental inflammation (placentitis)	Maternal immune response triggers infiltration of immune cells and cytokines into placental tissue.	IUGR, preterm birth, and potential neurodevelopmental issues due to reduced oxygen/nutrient supply.
Placental thrombosis	Infections can lead to blood clot formation within the placenta, reducing blood flow.	Hypoxia, miscarriage, stillbirth, preeclampsia, and fetal growth restriction.
Placental insufficiency	Placenta fails to deliver adequate nutrients and oxygen to the fetus due to infection.	Low birth weight, developmental delays, neurodevelopmental impairments, and preterm birth.
Viral transmission across placental barrier	Certain infections may directly cross the placental barrier, infecting the fetus.	Congenital infections (e.g., rubella, CMV, and Zika), developmental abnormalities, hearing loss, and neurological disorders.
Altered placental vascularization	Infections may disrupt normal placental blood vessel development.	Fetal hypoxia, preeclampsia, preterm labor, and complications in fetal growth.

**Table 2:** Summary of the various impacts of maternal COVID-19 infection during pregnancy on different aspects of the newborn’s development and health, highlighting both immediate and long-term concerns.

Area of Interest	Explanation	Potential Consequences
Neurological development	Maternal COVID-19 can lead to the release of inflammatory cytokines, which may cross the placental barrier and affect the fetal brain, especially in early pregnancy.	Altered neurodevelopment and risk of cognitive and behavioral delays such as language acquisition, motor skills, ASD, and ADHD; may not manifest until later childhood or adolescence.
Long-term cognitive and behavioral effects	Exposure to inflammation during brain development may result in structural and functional changes, affecting cognitive and behavioral health.	Increased risk of cognitive delays (e.g., memory, and attention), learning difficulties, and behavioral disorders such as ASD and ADHD.
Immune system development	Inflammatory markers passed from mother to fetus may affect the newborn’s immune system development.	Potential hyperactive immune response, increased risk of autoimmune diseases, or an underdeveloped immune system, leading to vulnerability to infections in neonatal period and beyond.
Placental dysfunction	COVID-19 may cause inflammation and thrombosis in the placenta, reducing oxygen and nutrient delivery to the fetus.	IUGR, hypoxia, increased risk of neurological complications, preeclampsia, preterm delivery, and possible fetal death.
Preterm birth and birth complications	Maternal COVID-19 is associated with higher risks of preterm delivery, which can impact brain development, particularly in the third trimester.	Cognitive delays, learning disabilities, difficulties with executive functioning, and increased risk of IVH and PVL.
RDS	Preterm birth increases the risk of RDS due to underdeveloped lungs, with maternal inflammation further complicating lung development.	Difficulty in breathing at birth, long-term respiratory issues, and higher risks of RDS and lung complications in the neonatal period and childhood.

## Data Availability

The information is contained within this article in its entirety. For additional information, please feel free to query either the original author or the corresponding author. Public access to the data is restricted as a result of the patient privacy standards that regulate the handling of clinical data.
